# Building Dynamic Capabilities and Organizational Resilience in Tourism Firms During COVID-19: A Staged Approach

**DOI:** 10.1177/00472875231164976

**Published:** 2023-03-31

**Authors:** Girish Prayag, Yawei Jiang, Mesbahuddin Chowdhury, Muhammad Ismail Hossain, Nasrin Akter

**Affiliations:** 1 Department of Management, Marketing and Tourism, UC Business School, University of Canterbury, Christchurch, New Zealand; 2 Department of Tourism, Sport and Hotel Management, Griffith Business School, Griffith University, Brisbane, QLD, Australia; 3 Department of Marketing, University of Dhaka, Dhaka, Bangladesh

**Keywords:** dynamic capabilities, organizational resilience, crisis management, COVID-19, planned resilience, adaptive resilience

## Abstract

Using dynamic capabilities (DCs) and the disaster/crisis management cycle (DMC) as the theoretical lens, this study explores how different types of DCs build and sustain organizational resilience of tourism firms during COVID-19. Taking a processual view, the study advances theorization of the relationship between DCs and organizational resilience in tourism studies. A qualitative study of 30 owners and senior managers of tourism and hospitality firms in Bangladesh reveals that threats and opportunities presented by the COVID-19 pandemic activated 10 different types of DCs (replicating, integrating, reconfiguring, creating, developing, assimilating, renewing, adaptive, innovative, and regenerative) across the pre, response (short-term) and future recovery intentions (long-term) stages. DCs activated different resilience facets (networks and relationships, leadership and culture, and change ready), highlighting the criticality of achieving planned and adaptive resilience for tourism firms during COVID-19. Response and recovery implications for tourism firms during disruptive events are suggested.

## Introduction

Crises and disasters have profound impacts on tourism firms. A policy brief by the United Nations World Tourism Organization (UNWTO) in 2020 on the COVID-19 pandemic highlighted that 80% of tourism firms worldwide are micro, small, and medium sized enterprises, with the economic fallouts from the global crisis affecting significantly tourism in developing and transition economies where government support for financial packages and social protection was insufficient (UNWTO, 2020). One of the recommendations was for the tourism industry to boost its competitiveness by becoming more resilient (UNWTO, 2020), requiring tourism firms to improve their resilience. Organizational resilience has received significant attention in several fields (Hillmann & Guenther, 2021; Raetze et al., 2022), including tourism (Hall et al., 2018; Prayag, 2020; Prayag et al., 2020; Wieczorek-Kosmala, 2022). Described as a capacity, process, strategy, trait, and outcome, or a combination of these (Hillmann & Guenther, 2021), the concept has no single accepted definition. There is also no consensus on the internal and external drivers of organizational resilience (Jia et al., 2020). In this study we utilize Jia et al.’s (2020, p. 10164) definition, which suggests that resilience is an “organization’s ability not only to develop preventive capacity to face any unexpected disruptions (i.e., planned) but also to take the necessary and quick actions to respond and recover from that disruption (adaptive) to ensure business continuity.” As a developable capacity, resilience can be nurtured in organizations (Hillmann, 2021) and deployed at different crisis stages using planned (proactive) and adaptive (reactive) elements. In particular, we adopt the disaster/crisis management cycle (DMC) in tourism of pre-crisis/disaster, during crisis/disaster (short-term) and post-crisis/disaster (long-term) (Faulkner, 2001; Jiang et al., 2021a), albeit we are focusing on future long-term recovery options in this study.

While dynamic capabilities (DCs) can allow tourism firms to manage crises and disasters, many struggle to identify or activate them (Jiang et al., 2021a, 2021c). Jiang et al. (2021c) call for research investigating how DCs can be developed and transformed into organizational resilience, despite an emerging research strand examining DCs in tourism firms during crises and disasters (Alonso-Almeida et al., 2015; Jiang et al., 2021a, 2021c; Shrestha & L’Espoir Decosta, 2023). Unfortunately, the same factors that build DCs have also been used as enablers of organizational resilience, leading to the latter being described as a type of DC (Corrales-Estrada et al., 2021). Using the DCs dimensions of sensing, seizing, and transforming and the DMC, Jiang et al. (2021b) identify how DCs can enable organizations to develop resilience, arguing that a process view of resilience creates a better alignment with the DMC. While Jiang et al. (2021b) provide a clear understanding of the role of DCs in achieving adaptive capacity, vulnerability reduction, situation awareness, and management of disruptive changes as part of building resilience at different stages of the DMC, their study does not consider other types of DCs beyond sensing, seizing and transforming. In a subsequent study, Jiang et al. (2021a) call for a broader view of DCs in tourism studies, providing a typology of different types of DCs using the DMC, but then do not identify the resilience outcomes. Responding to the two different calls by Jiang et al. (2021a, 2021c) for the integration of resilience in an understanding of DCs and the role of different types of DCs at various stages of crises and disasters, we evaluate how different types of DCs contribute to organizational resilience during the COVID-19 pandemic by focusing on planned and adaptive resilience elements. In particular, we consider adaptive elements of leadership and culture, network and relationship, and change ready (planned) elements of organizational resilience (Lee et al., 2013; Seville, 2018; Vargo & Seville, 2011). The planned and adaptive elements have received some attention in tourism studies (M. Chowdhury et al., 2019; Prayag et al., 2018, 2020; Sobaih et al., 2021), with the network and relationship building facet investigated under social capital (Bhaskara & Filimonau, 2021; Jia et al., 2020) or tourism networks (van der Zee & Vanneste, 2015).

**Figure 1.**
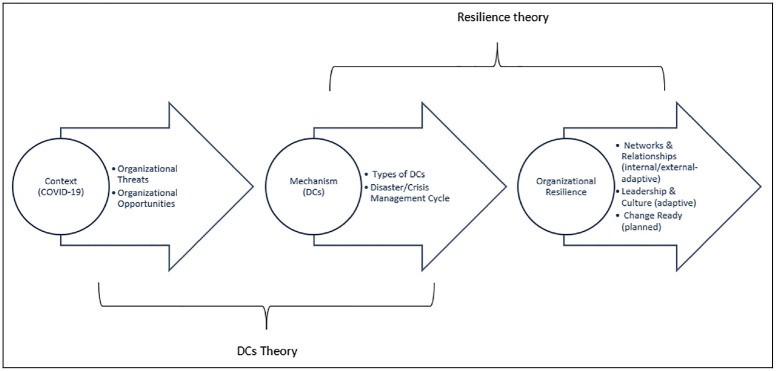
Theoretical framework of the study.

The call for greater consideration given to *context* (Raetze et al., 2022) and *mechanism* (Do et al., 2022) underpinning the development of organizational resilience, allows us to ground this study in middle-range theorizing (MRT) (Merton, 1968), which aims to understand and predict phenomena by focusing on the specific generative causes (or mechanisms) that produce outcomes in a particular context (Pawson & Tilley, 1997). In understanding how resilience develops in organizations, linkages between triggers, including their severity, direct and indirect impacts, as well as expected consequences must be studied (Raetze et al., 2022). Accordingly, Figure 1 describes the use of two grand-theories, resilience and DCs, to frame our understanding of how COVID-19 generates organizational threats and opportunities for tourism firms in Bangladesh, thus addressing the first element of MRT (*context*). From this understanding, we determine the types of DCs employed by tourism firms at different stages of the pandemic (*mechanism*) to achieve organizational resilience (*outcome*) in the three areas of networks/relationships, leadership and culture, and change readiness. Thus, all three elements of MRT (*context, mechanism, and outcome*) are evidenced in our theorization leading to two main research questions:

*RQ1*: What types of DCs were activated by tourism firms from the impacts (threats and opportunities) of the pandemic?
*RQ2*: How did DCs enable the development of organizational resilience at different stages of the pandemic?

The aim of this study, therefore, is to explore how different types of DCs build and sustain organizational resilience of tourism firms during the COVID-19 pandemic. Given that existing studies have either linked DCs of sensing, linking, and reconfiguring to tourism firms (Shrestha & L’Espoir Decosta, 2023) as a pandemic response or to their disaster resilience (Jiang et al., 2021b), we extend this literature by uncovering how the COVID-19 pandemic is shaping the resilience response of tourism firms in a less developed country using the DMC. This allows for the identification of resilience benefits extracted by tourism firms from DCs (Jiang et al., 2021b). From a theoretical perspective, we extend DCs theory by linking specific types of DCs to planned and adaptive resilience of tourism firms. We also use MRT to isolate opportunities and threats on tourism firms and their subsequent influence on DCs and organizational resilience. In this way, we extend the emerging research strand examining COVID-19 impacts on tourism businesses (Kuščer et al., 2022; Ntounis et al., 2022) by demonstrating the specific knowledge-based DCs (e.g., regenerating, assimilating, adaptive, and innovative etc.) that are activated as a result of the threats and opportunities presented by the pandemic. Thus, our findings bridge the knowledge gap in existing studies (Jiang et al., 2021a, 2021b) where only DCs of sensing, seizing and reconfiguration have been linked to disaster resilience.

## Literature Review

### COVID-19 Pandemic and Tourism Firms

The COVID-19 pandemic has disrupted several sectors, including tourism, often requiring quick and radical responses (Shrestha & L’Espoir Decosta, 2023), with mid- and long-term consequences uncertain (UNWTO, 2020). Yet, there is an expectation that long-term structural changes will occur in the industry (Sigala, 2020). The continuous need for economic support through government stimulus and intervention packages for the tourism industry indicates the crisis severity (Sharma et al., 2021; Sigala, 2020). Many tourism businesses are eager to go “back to normal,” accepting these economic support packages without considering their fairness, effectiveness and distribution (Higgins-Desbiolles, 2020). Alongside, support for tourism businesses in less developed countries has been sporadic (UNWTO, 2020). Several studies note the lack of research on pandemic impacts and opportunities for tourism firms (Kuščer et al., 2022; Price et al., 2022).

Existing studies suggest that plummeting travel confidence, border closures and changing visa requirements have created challenges for tourism businesses, highlighting the unpredictability of government responses to the pandemic (Gössling et al., 2021; Sigala, 2020). Operational aspects have been the hardest hit, including supply chain, finance and human resources management, causing loss in revenue and income, liquidity issues, and postponed or suspended business development plans (Arbulú et al., 2021; Gössling et al., 2021). Other tourism businesses have lessened their operational capacity through staff layoff, leading to rising unemployment in the industry (Baum et al., 2020; Kaushal & Srivastava, 2021). Skilled workers from the hospitality and tourism industry have also moved to other industries (Baum et al., 2020). Businesses have responded by multi-skilling employees, improving hygiene and sanitation standards, utilizing cash reserves, and boosting employee morale (Kaushal & Srivastava, 2021). While building psychological capital and using corporate social responsibility activities have been suggested as pandemic responses for tourism firms (Mao et al., 2021), others have engaged in relationship management with government, travel intermediaries and customers for recovery purposes (González-Torres et al., 2021).

The pandemic has also presented several opportunities for tourism businesses to “grow back better” (UNWTO, 2020) and improve their sustainability achievements (Gössling et al., 2021; Shrestha & L’Espoir Decosta, 2023). Terms such as “innovation” and “transformation” have been invoked as managerial ideals for business change during the pandemic (Price et al., 2022). Studying, micro and small firms in the UK, Price et al. (2022) found that those with good social networks adapted better and were more capable of extending their business models during the pandemic. They sensed their way forward using more informal information sources, while making some formal plans, but fundamental business changes were largely improvised, provisional, experimental, and contingent (Price et al., 2022). Thus, a transformation journey for these firms was largely inexistent. Existing studies on pandemic threats and opportunities for tourism firms are largely conceptual or focused on one impact type (e.g., supply chain, finances, and demand loss) or from western contexts. Yet, emerging evidence suggests that pandemic response and recovery strategies from tourism firms were built on internal and external capabilities. Organizations tend to rely on both types of capabilities during crises and disasters (Jiang et al., 2021a). Next, we examine how DCs can facilitate organizations to navigate crises and disasters.

### DCs Theory and Tourism Firms

DCs theory has evolved primarily as an integration of three school of thoughts (resource-based view-RBV, evolutionary theory of firms-ETF, and knowledge-based view-KBV). RBV focuses on competitive advantage derived from internal resources (Boxall, 1996). ETF argues that DCs are embedded in organizational processes that facilitate learning, guiding the evolution of DCs over time to respond to both short-term and long-term changes (Nelson & Winter, 1982). The most recent, KBV, argues that simply acquiring resources and capabilities may not be sufficient for organizational competitiveness (Eisenhardt & Martin, 2000; Li & Liu, 2014; E. Wang et al., 2007). Organizational knowledge is necessary to facilitate the recombination of existing capabilities (Bueno et al., 2008). Tourism studies have specifically employed Teece et al.’s (1997, p.156) definition of DCs as “the firm’s ability to integrate, build and reconfigure internal and external competencies to address a rapidly changing environment” for evaluating sensing, seizing, and reconfiguring capabilities (Jiang et al., 2019).

While existing studies view DCs as strategic, organizational, and managerial processes that create value for firms in dynamic markets by manipulating resources into new value-creating strategies (Eisenhardt & Martin, 2000; Teece et al., 1997), there is no consensus on the specific internal and external factors driving the development of DCs (Schilke et al., 2018). Factors such as firm experience, culture, resource access, leadership, external uncertainty, and readiness for change can facilitate their development (Schilke et al., 2018), indicative of organizations building on existing resources to develop new resources (Eisenhardt & Martin, 2000). Others suggest that access to existing external resources and co-developing new resources can create competitive advantage (Denford, 2013). Under the KBV, an organization’s absorptive capacity when facing disruption comprises of four organizational routines: acquisition, assimilation, transformation, and exploitation of knowledge (Zahra & George, 2002). This is essential in crises and disasters as knowledge-based resources are easier to activate and reconfigure than physical resources (Jiang et al., 2021a). Yet, internal process restrictions and external over-regulation can act as major barriers to the development of DCs (Jiang et al., 2021c).

### Organizational Resilience and Tourism Firms

Resilience can be a positive resource for achieving organizational outcomes (Hartwig et al., 2020). Integrating organizational resilience research across different fields, Raetze et al. (2022) identify three categories of resilience (developmental, proactive, and reactive). The proactive element can be equated to planned resilience where the organization improves its preparedness and readiness to disruptions in the short-term (Lee et al., 2013; Prayag et al., 2020) but requires a systematic investment in resilience building resources and capabilities in the longer-term to effectively cope with adverse future situations (Raetze et al., 2022). This calls for organizations to develop a *proactive posture* in relation to “strategic and behavioral readiness to respond to early warning signals from the internal or external environment before they escalate into a crisis” (Lee et al., 2013, p. 34). The proactive element also requires organizations to understand *internal and external resources* available and accessible through networks and relationships before a crisis (Vargo & Seville, 2011). As part of preparedness, *stress testing of plans* and staff simulations designed to practice response and mitigation strategies are critical (Lee et al., 2013). The *development and evaluation of plans* and strategies to manage key vulnerabilities in relation to the business environment and key stakeholders are seen as critical for response and recovery (LLee et al., 2013; McManus et al., 2008). In summary, these elements reflect the ***change ready*** facet of the organization and are essentially pre-disaster/crisis activities (Prayag et al., 2020). Generally, tourism firms struggle the most with preparedness aspects of resilience (Orchiston et al., 2016) suggesting that their change readiness can be low.

The adaptive capacity, also called adaptive resilience of the organization represents the short-term reaction to threats and crises (Raetze et al., 2022). It reflects that ability to bounce back, progress and create new opportunities from the unexpected (Lengnick-Hall et al., 2011). Drawing on McManus et al. (2008) organizational resilience model, adaptive resilience is activated during and post-disaster through indicators such as leadership and culture, staff engagement, decision-making, situation awareness, and innovation and creativity. Strong *leadership* that provides good strategic direction and decision making in disruptive times is essential for resilient organizations (Lee et al., 2013), often emerging as critical for the survival of tourism firms (Dahles & Susilowati, 2015; Orchiston et al., 2016). *Staff engagement* as displayed by behaviors such as an understanding of the link between their own work and organizational resilience, effective use of internal and external resources, and the ability to problem solve using existing skills are essential to the organization’s adaptive capacity (Lee et al., 2013). Related to this, staff that are empowered to make decisions related to their work are better able to also delegate responsibility and authority to enable a crisis response (Lee et al., 2013), highlighting the importance of *decision-making* abilities. Organizations that have a deeper understanding and perception of their operating environment in a disaster can look forward to opportunities, and forecast consequences accurately, facilitating their ability to bounce back (McManus et al., 2008). Thus, *situation awareness* becomes necessary as part of the organization’s adaptive capacity (McManus et al., 2008) but can also inform the proactive element for future disruptions. These factors in essence demonstrate the ***leadership and organizational culture*** that drives how disruptions are recognized and handled within an organization.

Finally, organizational resilience hinges on the ability to leverage the knowledge and resources embedded in internal and external ***networks and relationships*.** In particular notions of internal and external social capital are relevant to the ability of organizations to leverage knowledge during disasters (M. Chowdhury et al., 2020) and pandemics (Ai & Peng, 2021). *Breaking silos* through minimization of social, cultural and behavioral barriers in networks (internal and external) is considered critical to the ability to *leverage knowledge and resources* in disruptions (Prayag et al., 2020). The existence of silos contributes to communication barriers that are detrimental to collaboration and cooperation during crises (Lee et al., 2013). For *effective partnerships* to develop that can provide or improve access to external resources and knowledge during a crisis, organizations need to develop relationships based on trust, reciprocity and mutual understanding (M. Chowdhury et al., 2019). The importance of networks and relationships as one of the building blocks of an organization’s response and recovery from adversity has been acknowledged in the organizational resilience literature (Hillmann, 2021; Prayag, 2020). As such, networks and relationships are important for developing both planned and adaptive elements of resilience (Prayag et al., 2018; Sobaih et al., 2021).

### DCs and Organizational Resilience in Crises/Disasters

Studies linking DCs and organizational resilience are new (Jiang et al., 2019, 2021b). As a dynamic construct, organizational resilience is dependent on both operational and strategic resources such as leadership and change management (Hartwig et al., 2020). Building resilience across different types of adverse situations requires a mix of routine responses but also more flexible and innovative responses for strategic reconfiguration (Manfield & Newey, 2018). DCs can be viewed as the advanced mindset/strategy directions that flow into organizational management processes during crises/disasters that build resilience. Yet, there is no consensus on different types of DCs that allow organizations to build resilience in challenging environments.

Given that the magnitude and impact of disruptions vary, there is also no consensus on types of DCs required at different stages of the DMC to activate and build planned and adaptive resilience. A recent study grounded in KBV (Jiang et al., 2021a) identified 12 types of disaster related DCs to understand how organizations can navigate the different stages of the DMC. Table 1 provides a summary of these DCs. In the pre-crisis stage, DCs can enable strategy and actions that reduce vulnerability, mitigate impact, and improve performance (Jiang et al., 2021a). Thus, **replicating** and **integrating DCs** focus on leveraging existing and new resources internally in crisis response and recovery (Jiang et al., 2021a; Senivongse et al., 2019) to build planned resilience (see Table 1). Having a crisis management plan in place, for example, would identify redundant resources that can be replicated and integrated with other capabilities to respond. Integrating DCs, which include the use of scenario planning, ability to detect and forecast, and improve crisis/disaster readiness (Jiang et al., 2021a), are all pre-crisis activities that can boost planned resilience. Thus, replicating and integrating DCs can build the proactive posture of the organization, indicative of the change readiness facet of organizational resilience.

**Table 1.** Types of DCs, DMC and Organizational Resilience.

Types of DCs	Definition	DMC	Examples	Organizational resilience indicators
*Replicating*	Represents the internal exploitation of resources with the organization to prepare for potential disruptions (Jiang et al., 2021a; Senivongse et al., 2019)	Pre-crisis/disaster	Disaster management plan, safety kit, informal/formal operating procedure manuals	Adaptive Resilience (Networks & Relationships) *Information and knowledge
*Integrating*	Reflects the capability to recognize and explore new resources to integrate knowledge within the organization (Jiang et al., 2021a; Verona & Ravasi, 2003). It is the exploration of new knowledge through the recognition, transfer, absorption and application of internal knowledge into new organizational activities (Denford, 2013).	Pre-crisis/disaster	Safety drill rehearsal, practice evacuation procedures, scenario planning tools	Planned resilience (Change Ready) *Proactive posture *Planning strategies
*Reconfiguring*	Coordinates the accumulation of knowledge by combining, extending and exploiting pre-existing organizational know-how from operational processes and businesses to build bundles of knowledge (Helfat, 1997). Organizations can respond to unexpected changes quicker and in flexible way (Jiang et al., 2021a).	During and post-crisis/disaster (short-term)	Changing communication channels, advertising/messaging content	Adaptive resilience (Leadership & Culture) *Leadership *Staff engagement and involvement (Networks & Relationships) *Internal resources Planned resilience (Change Ready) *External resources *Recovery priorities *Planning strategies
*Creating*	Reflects the use of a firm’s internal exploration for new knowledge through combining existing resources and knowledge base (Denford, 2013). Organizations can quickly modify markets and products to survive (Jiang et al., 2021a).	During and post crisis/disaster (short-term)	Modify target markets, products and services, diversification, culture of experimentation and innovation	Adaptive resilience (Leadership & Culture) *Leadership *Decision making Planned resilience (Change Ready) *Recovery priorities *Planning strategies
*Developing*	Generates new knowledge outside the firm through recombination of firm’s own and partners’ knowledge (Denford, 2013).	During and post crisis/disaster (short-term)	Relationship building activities within a sector, stronger linkages to government and wider communities	Adaptive resilience (Leadership & Culture) *Staff engagement and involvement Planned resilience (Change Ready) *Planning strategies
*Assimilating*	Involves external exploration for new knowledge to be absorbed into the firm (Denford, 2013). This requires processes to analyze, interpret and understand external information (Zahra & George, 2002). Organizations can accurately sense and recognize useful resources available from external partners (Jiang et al., 2021a).	During and post crisis/disaster (short-term)	Collaborative market research and opportunities identification	Planned resilience (Change Ready) *External resources *Planning strategies
*Adaptive*	Represents the organization’s ability to strategically link existing resources to the new and changing environment (C. L. Wang & Ahmed, 2007). The organization can use this DC to exploit existing resources to capitalize on the new and emerging opportunities (Jiang et al., 2021a).	Post-disaster (long-term)	Upgrade of infrastructure and assets, human resource development strategies	Adaptive resilience (Leadership & Culture) *Staff engagement and involvement Planned resilience (Change Ready) *External resources *Recovery priorities *Planning strategies
*Renewing*	Organization’s capabilities to refresh and augment the resource base or generate new capabilities (Jiang et al., 2021a)	Post-disaster (long-term)	Continuous business reinvestment and resources augmentation	Adaptive resilience (Leadership & Culture) *Staff engagement and involvement (Networks & Relationships) *Minimization of silos Planned resilience (Change Ready) *Planning strategies
*Regenerative*	Exploitation of external resources to benefit internal operations without changing business structure and culture (Jiang et al., 2021a)	Post-disaster (long-term)	Hiring new directors/CEOs, forward planning,	*Adaptive Resilience* (Network & Relationships) *Situation monitoring and reporting Planned resilience (Change Ready) *External resources
*Innovative*	Enables organizations to shift to more transformational organizational change (Jiang et al., 2021a). This implies a change in business structure, culture and organizational design (Teece, 2007).	Post-disaster (long-term)	New business collaborations, new ventures, internationalization strategies.	*Adaptive Resilience* (Network & Relationships) *Innovation creativity

During the crisis or disaster, both absorptive and adaptation capabilities are required, and thus resourcefulness, adaptability, and flexibility become critical (Conz & Magnani, 2020). **Reconfiguring DCs** that improve flexibility (Jiang et al., 2021a) through boosting the ability to combine, extend and exploit organization know-how (Helfat, 1997) would allow organizations to leverage their situation awareness and fast decision-making capabilities to respond (Lee et al., 2013). In essence, these are activities that contribute to adaptive resilience. Also, **assimilating DCs**, for example, can facilitate organizations to sense and recognize information available from external networks (Jiang et al., 2021a), and when such capabilities are integrated with the existing organizational know-how (reconfiguring DCs), the response and recovery are based on adaptive resilience that demonstrates situation awareness and the use of innovation and creativity. This process reflects absorption capabilities of the organization, requiring redundancy, robustness and agility as core capabilities (Conz & Magnani, 2020; Corrales-Estrada et al., 2021) to achieve resilient outcomes. **Creating DCs** through existing knowledge management and resources allow organizations to quickly modify markets and products (Denford, 2013; Jiang et al., 2021a). By leveraging staff engagement and fast decision making (Lee et al., 2013), adaptive resilience hinges on how well these creating DCs are activated in the crisis response strategy. External networks help to acquire and transform resources, which reflect the essence of DCs (Blyler & Coff, 2003). **Developing DCs** allow organizations to capitalize on external networks (Denford, 2013) to respond to disasters using external information, knowledge and resources. The benefits emanating from these capabilities facilitate the organization to break silos, form effective partnerships and leverage external knowledge (Lee et al., 2013) in disaster response and recovery strategies. Thus, developing DCs facilitate organization to use networks and relationships to boost adaptive resilience.

DCs can also in the long-term help organizations to maintain business continuity and sustain growth post-disaster (Jiang et al., 2021a). DCs can provide the impetus for substantial organizational change (e.g., changing strategic direction), transformation and diversification opportunities (Jiang et al., 2021a). They can also improve cooperation, networks and relations, knowledge management, and strategic human resources management (Dyduch et al., 2021). Thus, **adaptive DCs** that strategically link existing resources of organizations to capitalize on new and emerging opportunities post-disaster hinge not only on leadership qualities, situation awareness and unity of purpose in long-term response but also proactive posture (see Table 1). Battisti and Deakins (2017) link proactive posture, which indicates an organization’s behavioral readiness to the adoption, creation and acquiring of new capabilities and resources to the ability of organizations to respond to changes in the external environment. Thus, both planned and adaptive resilience in the long-term hinge on adaptive DCs that are activated post-disaster to maintain business continuity and the investment strategies of the organization to boost resilience capacity over time. In the long-term, organizations can also use external resources to benefit internal operations (Jiang et al., 2021a) and engage in developing capabilities that showcase forward planning. Thus, **regenerative DCs** that continue to leverage external knowledge, break silos and reinforce existing partnerships can boost both planned and adaptive resilience. DCs can improve the ability to innovate and imitate, move resources quickly, use new technology (Dyduch et al., 2021), delegate power and create greater autonomy of employees, while also boosting creative idea generation and bottom-up experimenting (Dyduch et al., 2021). These benefits emerge from **innovative DCs** that can enable organizations to undertake a more long-term transformation by revisiting organizational culture, structure and design (Teece, 2007). These benefits can also have flow on effects on unity of purpose, leadership, and planning strategies of organizations (McManus et al., 2008), thus enhancing both planned and adaptive resilience.

## Methods

### Research Context—Tourism in Bangladesh and COVID-19

Bangladesh was chosen as the location of the study for two reasons. First, prior to the pandemic, that is, in 2019 Bangladesh was the third fastest growing economy globally with a GDP of over $409 billion (United Nations, 2019). The size of its economy was forecasted to double by 2030 and expected to become the 24th largest economy in the world by 2036 (Ahmed, 2021; CEBR, 2021). Domestic demand was expected to be driven by a fast-growing and large middle class with a GDP per capita of US$ 6,019.52 in 2021 (Hussain et al., 2020; Trading Economics, 2022). This was expected to create an even higher demand for lifestyle products such as tourism (Munir et al., 2015). However, as outlined in the next paragraph, the industry was severely impacted by the COVID-19 pandemic. Second, the economic promise and high demand for tourism products (both outbound and domestic) make Bangladesh an attractive destination for local and international tourism marketers despite of COVID-19. In particular, significant domestic investment has been made in the tourism sector (S. Hossain, 2019; Uddin, 2017). However, the COVID-19 pandemic has created a business environment that challenged the survival of tourism organizations (Haque, 2021) but the long-term expansion of the industry is still expected.

Bangladesh’s response to COVID-19 was also unique in that simultaneous response and recovery were enacted. For example, the tourism industry was already focused heavily on the domestic market before the pandemic, and there were no significant or strictly observed lockdowns enforced in Bangladesh in comparison to Western countries such as Australia and Europe (Biswas et al., 2020). Thus, the response stage was very short while the recovery stage started earlier than many other countries. In 2019, the tourism industry contributed 2.7% to the GDP of Bangladesh (WTTC, 2021). Domestic tourists are the key drivers of this industry rather than international visitors. Since the beginning of 2020, the industry has been severely impacted by the pandemic (E. K. Chowdhury, 2020). All economic data (e.g., GDP, employment opportunity) show a sharp decline—with the tourism industry’s contribution to GDP dropping by 32.9% (from 2.7% in 2019 to 1.7% in 2020) (WTTC, 2021). Similarly, its contribution to overall employment dropped by 21.9% (from 1.86 million in 2019 to 1.45 million in 2020) (WTTC, 2021). Considering tourism as an emerging industry that can contribute to the national economy, the government of Bangladesh had allocated BDT 34 billion in 2019 to 2020 through the Civil Aviation and Tourism Ministry for the development of this sector prior to the pandemic. To mitigate the impact of COVID-19, the government of Bangladesh implemented a stimulus package of BDT 50 billion for export-oriented industries that did not include the tourism industry (KPMG, 2020). Nonetheless, the government consulted with different stakeholders to develop a plan for the tourism sector to benefit from the stimulus package and low interest-bearing bank loans that will allow the sector to survive and bounce back (Sakib, 2021).

### Interview Protocol

From the outset, potential participants were recruited through an information sheet, seeking their consent for participation. A further qualifier question identified whether their tourism business was impacted by the COVID-19 pandemic, and a negative response led to their exclusion from the study. From the existing literature, we developed a semi-structured interview protocol (see Appendix A) that focused, first, on understanding the impacts of COVID-19 on the tourism industry in Bangladesh and the participant’s organization. Questions, which included probing, were adapted from previous studies (Foo et al., 2021; Yeh, 2021). Next, the interview focused on different response strategies and practices used by participants’ organizations during the pandemic (Yeh, 2021). To get further insights, we probed on areas such as staff engagement, networks and relationships with other businesses, and leadership and organizational culture, drawing on suggestions in previous studies (Fang et al., 2020; Su et al., 2021). Further questions focused on organizational adaptation and stability (Fuchs, 2021) and participants were asked about organizational practices they believed contributed to resilience (Fang et al., 2020). We also probed on areas such as new processes and initiatives introduced during the pandemic.

We pre-tested the interview protocol on three tourism industry experts in Bangladesh. The first expert was a senior academic from a leading public university, who is an active researcher in the field of tourism and hospitality. The other two experts were CEOs of tourism organizations in Bangladesh with substantial industry experience. All interviews were carried out during the first wave of the pandemic (i.e., April–May 2020) when the country was partially in lockdown. As COVID-19 fear was prevalent, participants were reluctant for face-to-face interviews. Instead, all interviews were conducted on zoom (Kuščer et al., 2022). The original interview protocol was designed in English and translated into the local language (i.e., Bangla) by one co-author of this study. A second co-author (whose first language is Bangla and proficient in English) verified the translated version for translational equivalence (Brislin, 1970). All interviews were carried out in Bangla and back translated in English.

### Sampling and Data Collection

The target population for this study was defined as hospitality and tourism organizations active and operating at the time of data collection. Particularly, we selected key decision makers (i.e., CEO, Owners, or Managers) with the ability to undertake strategic decisions during the pandemic. To identify such participants, we first contacted the University of Dhaka, who opened the first Tourism and Hospitality Management department in Bangladesh back in 2007. The department maintained a list of graduates (alumni) who are currently working in the tourism industry. Using this database, we initially identified a list of 75 potential participants based on their strategic decision-making responsibilities (i.e., holding a senior management position within the organization) along with their contact details (i.e., email and mobile number). This fits with a purposive sampling process. We recruited three research assistants (RAs) to contact these participants through email and phone conversations, which led to a total of 25 participants initially giving their consent to participate. To further ensure the appropriateness of the selected participants, we followed two procedures. First, RAs visited the social media pages of these businesses to get an understanding of their recent posts, which indicated how active these organizations were during the pandemic. Second, RAs gave a further phone call to these organizations to check their operating status prior to the interview and were following operational guidelines provided by the government. These background checks ensured that selected participants were suitable. However, five who had initially gave their consent for participation withdrew from the study, leaving a sample size of 20 for the first phase of interviews.

To increase the number of participants, a second phase of interviews was conducted by one of the co-authors who contacted Tour Operators Association of Bangladesh (TOAB) to identify potential participants. From the TOAB database, a list of another 50 potential participants were identified following the same criteria as mentioned before. By adopting a similar purposive sampling procedure, the RA team got a positive response from another 12 participants. However, two participants later withdrew from the interviews. Data saturation principles were applied to determine sample size. Saturation is the point in the data collection when no new additional ideas or themes emerge (Jordan & Prayag, 2022). With the first 20 interviews, data saturation was not reached as new ideas kept emerging, which led to the second phase of interviews described above. Another 10 interviews were needed to reach saturation. Combining the two phases, 30 semi-structured in-depth interviews were carried out by one of the co-authors of this study. All interviews were conducted on Zoom, video recorded and transcribed. The average duration of the interviews was approximately 76 min, with a minimum and maximum duration of 55 and 117 min respectively.

### Data Analysis and Trustworthiness

This study used NVivo 12 to organize and analyze the verbatim interview transcripts, which included categorization and analysis of emergent concepts. Constant comparison was used to identify common categories to develop further themes. Following Shepherd and Williams (2014), three main data analysis steps were used to ensure robustness and trustworthiness in analyzing opportunities and threats of COVID-19 on tourism businesses (see Table 2). First, first-order coding was conducted to identify initial categories using descriptive phrases. Second, axial coding was conducted to organize first-order codes into theoretical categories (themes) by understanding linkages among the initial categories leading to higher-order categories. Last, themes were synthesized into aggregate theoretical dimensions of COVID-19 impacts on tourism businesses.

**Table 2.** Thematic Summary and Coding Results (Impacts).

Aggregated dimension	Theoretical categories	First-order codes	Example direct quotes
Operational Threats *(capacity, finance, human resources)*	Business Activity Suspension	Facilities/venues closure	*Currently I am running only 2 outlets out of 12 outlets, 10 outlets are shut down for various reasons (Interview #06)*
		Customer interaction loss	*The preparation to avoid the impact of Covid-19 relates to activities which are completely opposite to how we are offering services to our clients. It is a big challenge for us to get closer to the guest. (Interview #10)*
		Business activities postpone	*In the middle week of January, more bookings were cancelled due to the lockdowns imposed in Bangladesh during March 2020. (Interview #18)*
		Business capacity reduced	*From 26 March, we locked down our resort.* By *keeping only about 20% employees with limited service provided (Interview #14)*
		Outbound tourism minimized	*For aviation, our flights are totally closed and that causes the tourism sector to come to a standstill, especially the outbound flights. (Interview #24)*
		Social responsibility minimized	*We used to do some programs thinking not only about the business but also about our social responsibility. Those work will be minimized. (Interview #17)*
		Investment/development plan suspended	*Many hotels will be shattered and shut down though few hotels and organizations in the tourism sector will continue with low profit and develop gradually. There will be no new investment in the tourism sector. (Interview #07)*
	Financial Burden	Income/revenue loss	*The revenue is very low for outbound tour operators in Bangladesh due to extreme competition. It is doubtful whether I will be able to survive another 4-5 months with what I have earned in the last 11 years (Interview #24)*
		Operational costs non-sustainable	*There are 493 rooms in my hotel. For me, it is a matter of great concern if the percentage of occupancy of these rooms can be confirmed. I have to cover this fixed cost. (Interview #10)*
	Employment Crisis	Layoff and unemployment	*Huge job cut will be another major impact. Rate of early/ voluntary retirement will also be increased. New policy like “Restructuring & Redundancy policy of emirates” will be imposed and result in huge job loss. (Interview #08)*
		Labor leakage to other industries	*Employment is a big factor. We’ll lose skilled workers. Even if everything gets sorted, I think 70-80% workers will not get back to this sector again. (Interview #09)*
		Loss of skilled workers and their connections	*We have been trained employees for seven to eight years…Guests will also leave when the employee leaves. Because they have formed a good relationship. (Interview #18).*
Operational Opportunities *(procedure, innovation, and planning)*	Health Standard Improvement	Hygiene and health safety awareness	*There is no doubt that the aviation industry will put health and cleanliness at the forefront of their priorities. (Interview #26)*
	Product and Service Innovation	Cloud kitchen	*Cloud kitchen is basically a warehouse and is shared by multiple other brands…The traditional cost has completely shielded. We do not need a physical space on the roadside. Rather we need a platform like Food Panda, Hungry Naki etc. for food delivery… Our sales mixes are shifting from dining to delivery. (Interview #05)*
		Home-delivery service	*Due to the social distancing people are not being able to come to the restaurants, we found an opportunity like home delivery for foods. (Interview #04)*
	Forward planning	Learning for future readiness	*There will be a lot of learning outputs of covid 19 such as work for the best but be prepared for the worst…maybe the business pattern and many strategic changes would occur, but we can see some good things. (Interview #13)*
Market Threats	Market and Demand Loss	Travel tendencies and confidence reduced	*Maintaining the health norm at this time would be challenging both from the services provider and customer perspective. If we found a COVID-19 positive individual at Cox’s Bazar…no one would want to visit that place. (Interview #12)*
		Border closure and international travel restriction	*Now if we consider all 5-star hotels, local hotels, guest houses the ratio is 20-80. 20% hotels are continuing their operation on a limited scale and 80% hotels are closed and they are uncertain whether they will reopen and continue their operation. (Interview #30)*
		Tourists’ economic condition affected	*Industries like us who are related to luxury goods or services would be in a risky situation and we would need the most time to overcome this risky situation. (Interview #02)*
		Visa change	*Bangladesh has imposed restrictions on ‘arrival visa’. If it continues, people will not come from outside, there will be no flow of people. (Interview #28)*
Market Opportunities	Market Transformation	Regional and domestic tourism thriving	*We have explored various new segments in our business. These explorations will help us to make ourselves diversified. (Interview #29)*
Tourism System Threats	Supply-Chain Breakdowns	Supply chain interruption	*We have suppliers, poultry, beef, vegetables, their families are depending on us as well. So it’s a complete chain effect interconnecting with each other. All the chain would be badly affected. (Interview #13)*
		Transportation system shutdown	*All the travel related transportation systems are shut down. It is one of the biggest industries in the world contributing almost 10% of world* GDP. *Due to the outbreak of covid 19, we are affected badly. (Interview #01)*
Tourism System Opportunities	Tourism Sustainability Development	Future planning (environment and nature)	*Now we have an opportunity to add different steps to monitor and it will help us in future for self-protection and environmental protection too…This has created a place for us to work more in these [environmental] matters. (Interview #23)*
		Value and process review (industry and business)	*Maybe the Airlines will reschedule their old management system for next 20-30 years…What will happen then is, they will select the staff in a systematic and planned way and with minimum staff they can continue operation. (Interview #25)*
		Industry transformation (new form)	*After the end of the epidemic, our aviation sector will come out of the monopoly as it was before. There will be an opportunity to re-establish it. (Interview #26)*

Resilience indicators were analyzed following an inductive data coding and constant comparison approach and four steps were followed to ensure accuracy and trustworthiness. First, a three-dimensional interdependent resilience framework (leadership & culture, networks & relationships, and change ready) (Vargo & Seville, 2011) and its 13 indicators (Lee et al., 2013; Prayag et al., 2020; Sobaih et al., 2021) were used as the overall coding themes in three crisis management stages (crisis preparation, crisis response, and crisis recovery). Second, following Shepherd and Williams (2014), open coding was conducted to identify initial categories under each indicator (first-order). Third, axial coding was implemented to understand linkages among the first-order nodes (higher-order). Last, all identified first-order nodes were aligned with DCs typology by assessing its source of resources used and the deployment pattern (Jiang et al., 2021a) (see Table 3).

**Table 3.** Thematic Summary and Coding Results (Utilization of Dynamic Capabilities for Resilience Building).

Crisis management stages	Resilience indicators		Open and axial coding	Dynamic capability typology	Example direct quotes
Crisis Preparation (pre-crisis stage)	Network & Relationship	Information and Knowledge	Leveraging knowledge from previous experience	Replicating DC	*Nobody was prepared. But US-Bangla airlines had gathered experiences to face any disaster or crisis management…Since we had overcome those situations, our previous experiences would help us to move forward to the future. (Interview #21)*
	Change Ready	Proactive posture	Contingency plans making	Integrating DC	*We took the precautions in operation as people were coming from China. It was early-January or mid-January when Covid was not even found in Bangladesh. We used to measure the temperature of our guests, kept a log, and monitored them with our house doctor if they had a temperature. We had hand sanitizer at every entry point of our hotel. So, we had our preparation in. (Interview #02)*
		Planning strategies	Reorganizing products	Integrating DC	
	*No Preparation		Out of control in business crisis preparation	N/A	*I think people can’t prepare for that sort of crisis. You can take preparations for flooding, storms etc. But preparing for not earning money month after month is not possible. None of us experienced this type of crisis before. (Interview #11)*
			No expectation of the worldwide spread and magnitude	N/A	*We were unsure if this virus may affect our country. So* that is *why we did not take any constructive steps against this initially. This was truly an unexpected scenario for us. (Interview #01)*
			No expectation on long time impacts	N/A	*We consider these calamities may affect us for 40-45 days at best. So*, COVID-19 *has a bigger impact than our assumption, and therefore we are a bit tense for our further steps. (Interview #29)*
Crisis Mitigation (short-term response stage)	Leadership & Culture	Leadership	Cost-balancing	Reconfiguring DC	*Office rent has been managed by negotiating with the landlord. Considering the current pandemic, those of us who are at the chairman level are refraining from taking salaries for our business. (Interview #12)*
			Forward planning	Creating DC	*After the opening we should sit and find out what is our payroll, how much the overhead cost is. We should sit together with our CFO and find out how much money is required to survive in a month. (Interview #02)*
		Staff engagement and involvement	Staff remote connection	Reconfiguring DC	*We are conducting all our meetings online. We also had to go through numerous adaptations. (Interview #13)*
			Staff social and mental support	Reconfiguring DC	*We are conducting some CSR activities like creating funds for labor and mid-level employees who are not getting salary. We are trying to boost them up mentally as well. (Interview #08)*
			Staff online training	Developing DC	*We also suggest all our employees to take online training officially in their free time. Thus, they can develop themself. (Interview #01)*
		Decision making	Emergency team development	Creating DC	*We formed an emergency team of 19 people. Those will stay at the hotel and look after the hotel until the situation gets normal. (Interview #22)*
	Networks & Relationship	Internal resources	Product adaptation	Reconfiguring DC	*As people are free at home, we sell the frozen food. We see whether it attracts people. (Interview #11)*
			Staff retention and loyalty	Reconfiguring DC	*The employees will be kept based on their efficiency, loyalty and their importance to the organization. (Interview #04)*
	Change Ready	External resources	Customer relationship maintenance	Reconfiguring DC	*My sales and marketing knocked door to door and communicated with the clients differently. Now we are communicating with them through electronic media like WhatsApp, we are boosting up many things with them about awareness. (Interview #23)*
			Coordination and communication across partnerships	Assimilating DC	*To fix our problems, we are working together. Businesses that are associated with us are doing jobs for everyone’s betterment. Without coordination nothing can be possible. (Interview #20)*
			Employment fundings	Assimilating DC	*Our prime concern should be the betterment of our employees. All the decisions will be taken based on Govt. and NGOs funds. Funding resources may help us to fight against this situation. (Interview #01)*
		Recovery priorities	Operational costs reduction	Reconfiguring DC	*I am reducing the numbers of employees in my organization. We are shifting to a small office. Thinking of cutting the overall cost down. (Interview #09)*
			Revenue maintaining	Reconfiguring DC	*We don’t know what will happen tomorrow. We have to ensure that we are getting the basic revenue so that we don’t have to touch our savings. (Interview #04)*
			Domestic market adaptation	Reconfiguring DC	*Five-star hotels focus on the foreign nationalities. But my idea will be to bring more Bangladeshi people to our hotel. Up until a vaccine is coming, international travelling will not revive. (Interview #05)*
			Health monitoring system	Creating DC	*There are many more things to think about now, including regular and proper cleanliness, keeping staff health issues in mind which is also costly. (Interview #10)*
		Planning strategy	New partnership	Developing DC	*We got permission from the government to operate cargo flights. So, we were able to operate a non-schedule cargo flight from Dhaka to Bangkok the other day. We had another cargo flight to Kolkata several days before. (Interview #21)*
			New products and market shift	Creating DC	*Currently the biggest concern is safety, now we need to adapt this in our food preparation. Suppose the way I package my food is completely contactless, this could be a marketing tactic. (Interview #05)*
			Regular routine updates	Reconfiguring DC	*We updated the major operation routine in our kitchen to control the quality. For example, wearing gloves for delivery drivers, ensuring the food are double-checked. (Interview #05)*
			Technology reliance for less human interaction	Assimilating DC	*Now we do non-cash transactions. Payment is made through Food Panda-our biggest stakeholder. We are also considering the idea of home delivery. (Interview #11)*
Crisis Adaptation (future long-term recovery stage)	Leadership & Culture	Staff engagement and involvement	Human resources development	Renewing DC	*We are doing some personal development, professional development. (Interview #10)*
			Staff empowerment	Adaptive DC	*We usually discuss openly with the staff when making a decision so that they too can participate in our decision making. They worked with the utmost sincerity because they consider the organization as their own. (Interview #26)*
		Situation monitoring and reporting	Safety information building	Regenerative DC	*From the very beginning, safety information were communicated in different social media and internet. The government has also provided many instructions on health safety information on COVID-19. (Interview #30)*
		Innovation and creativity	Humanity service	Innovative DC	*Some of the services that we are rendering now is offering/arranging transportation/logistic services to foreign nationals who are stuck in Bangladesh. We are connecting them with different embassies and connecting with different chartered airlines so that they can go to their own country. We are doing this only for the sake of humanity (Interview #12)*
	Networks & Relationship	Minimization of Silos	Negotiating financial issues	Renewing DC	*To reduce our expenditure, we have talked with our property owner to curtail the monthly rent. I tried to renegotiate with all my vendors as well. Also, to face government policies, I am working with several associations. (Interview #20)*
	Change Ready	External resources	B2B partnership building	Regenerative DC	*We grow our business based on collaboration with other companies. There are 10-15 companies that give our 60% revenue. We are already in connection and contact with them. (Interview #17)*
			B2C communication and relationship building	Adaptive DC	*My team is also thinking about our business model and plan. If such software can be developed, one-to-one communication with the customer can be done through it, and the customer can be communicated more effectively. (Interview #10)*
		Recovery priorities	Meeting COVID rules for new normal	Adaptive DC	*We have to take requisite hygienic and sanitation measures. As a boutique hotel we always provide our customers personalized service, socializing with them, but now we hardly get to see our customers. In consequence our service pattern is changing. (Interview #30)*
			Prioritizing customer satisfaction to survive	Adaptive DC	*We will invest more to make our food quality even better. Instant cooking and serving, delivering immediately, answering all the queries instantly, these are the things we will focus on to rebuild our relationship with the customers. (Interview #05)*
		Planning strategies	Brand equity reinforcement	Renewing DC	*Due to COVID, the other customer segment would also shrink and we need to figure out the changed market size. Keeping this in mind we will have to bring back the confidence of our customer by bagging the reputation of our brand. (Interview #27)*
			Learning and product development	Renewing DC	*We are learning to work with product development and new product ideas. Now it’s time for us to rethink the products we’ve never looked at before. (Interview #16)*
			Domestic market promotion	Adaptive DC	*We are changing the composition of target market. We have seen that the Dhaka market campaign does not go well in the Chittagong market. Our main strategy now will be to set the domestic market. (Interview #10)*
			Service pattern (routines) updates for future events	Adaptive DC	*A proper SOP should be implemented. Every owner of a hotel should abide by these SOPs. A central committee should take place to monitor all these activities. We have to look forward to circulating all the businesses that are interlinked with hotel management. (Interview #20)*
	*No planning (waiting for change)			N/A	*We have not set any strategies, because until now we cannot understand what the condition would be after one month. But primarily we have started working out since the vaccine is coming out. (Interview #17)*

**Figure 2.**
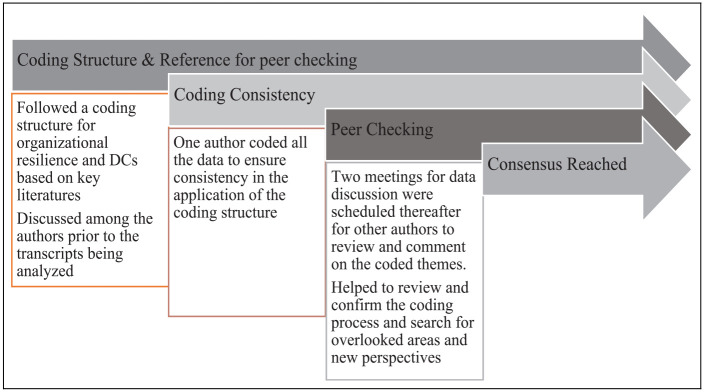
Trustworthiness process.

Several additional strategies were employed to ensure data trustworthiness at the coding stage (see Figure 2), using established techniques (Lincoln & Guba, 1985). Credibility of data interpretation was ensured given that three of the co-authors are from Bangladesh and have adequate knowledge of the local tourism industry and COVID-19 impacts on this industry. Data transferability was enhanced through the pre-design of the interview protocol, which was applied consistently throughout the data collection process. Also, a detailed investigation of participants’ personal information (e.g., educational background, career and working experience) complemented the comprehensiveness of data interpretation. Furthermore, trustworthiness was obtained through providing detailed contextual information and use of direct quotes and thick descriptions to convey the findings.

## Findings

### Sample Demographic and Business Characteristics

Table 4 summarizes participants’ demographics and characteristics of participating tourism firms. The average age of participants was 44.7 years. All participants were males, reflecting not only employment demographics in this industry but also a patriarchal society. These participants represented tourism and hospitality firms of various sizes, covering large (16), medium (4), small (5), and micro (2) (three participants did not disclose their firm size). In Bangladesh, the Industrial Policy of 2016 defines large service firms as having more than 120 employees, medium sized service firms having 51 to 120 employees, small service firms having 16 to 50 employees, and micro sized service firms having less than 15 employees (M. I. Hossain & Shah, 2016). The range of tourism and hospitality businesses included hotels (9), tourism (7), tourism and hospitality (5), aviation (5) and restaurants (4).

**Table 4.** Sample Summary.

Respondent	Role	Age	Sector	Firm size	Brand operation
1	Senior Manager of Marketing and Communication	36	Hotel	800+ (large)	International
2	Director Marketing and Business Promotion	42	Hotel	500–1,000 (large)	International
3	CEO	35	Tourism	10–12 (micro)	Local
4	General Manager	38	Hotel	200+ (large)	International
5	Head of Brands	30	Restaurant	300+ (large)	Local (1) + International (1)
6	Owner	36	Restaurant	250 (large)	Local (5) + International (1)
7	Director	66	Tourism and Hospitality	678 (large)	Local
8	Regional Head in Bangladesh & Nepal	39	Aviation	197 (large)	International
9	Managing Director	52	Tourism and Hospitality	9 (micro)	Local
10	Director of Sales and Marketing	37	Hotel	800+ (large)	International
11	Director	30	Restaurant	100–150 (large)	Local
12	Managing Director	49	Tourism	250 (large)	Local
13	Executive Director	69	Hotel	370 (large)	International
14	Head of Sales and Marketing	38	Hotel	400 (large)	Local
15	Owner	55	Tourism	60 (medium)	Local
16	Director	55	Tourism	17 (small)	Local
17	Assistant General Manager	36	Hotel	232 (large)	Local
18	CEO	37	Tourism	55 (medium)	Local
19	Managing Director	69	Tourism and Hospitality	N/A	Local
20	Executive Director	62	Tourism and Hospitality	24+ (small)	Local
21	General Manager, Public Relations Department.	48	Aviation	197 (large)	International
22	General Manager	36	Tourism	138 (large)	International
23	General Manager	43	Hotel	75–80 (medium)	Local
24	CEO	39	Tourism	40 (small)	Local
25	Marketing Manager	48	Aviation	17 (small)	International
26	CEO	36	Aviation	150 (large)	Local
27	Marketing Manager	38	Restaurant	40 (small)	International
28	Editor	67	Aviation and Tourism	N/A	Local
29	Deputy Managing Director	39	Tourism and Hospitality	N/A	Local
30	Operation Head	36	Hotel	62 (medium)	Local

First, the major themes for threats and opportunities of COVID-19 on tourism businesses are outlined, followed by the actions and practices that reflect different types of DCs used in a staged DMC approach to achieve organizational resilience. The three DMC phases and respective resilience goals are: preparation phase *(pre-crisis to build up preparedness)*, response phase *(short-term after crisis to mitigate negative impacts)*, and immediate as well as long-term recovery phase *(future long-term recovery to develop adaptive practices intentions)*.

### Threats of COVID-19 for Tourism Businesses

#### Industry Supply Chain and Destination Accessibility

First, due to flight restrictions, the supply chain of most businesses in this study was disrupted. The most apparent is the chain of *“airport, airline companies, travel agencies, and other related tourism service providers”* that faced dwindling demand during the pandemic. Pre-booked tours and accommodation cancellation followed from travel-related transportation system shutdown and destination inaccessibility. Furthermore, inaccessibility to markets disrupted the supply chain, which had flowed on effects on the accommodation industry, highlighting the interconnectedness of the hospitality and tourism industry with other industries, which indirectly led to operational costs rising.

#### Market Demand Loss

Declining market demand was the biggest negative impact of COVID-19 on tourism businesses as reflected in these three factors. First, international and domestic visitor numbers fell due to border closure and travel restrictions. Second, visitors’ travel behavior and confidence had shifted due to increased perceived travel risk (e.g., fear of virus), and precautionary measures (e.g., hygiene requirements). Third, tourism was sometimes considered a “luxury” for people who were struggling to survive, thus they reallocated tourism expenditure to daily living requirements:

…Now the number of family tours will decrease substantially as many families’ economic condition is not good at the moment…people are sitting at home and have no source of income. (Interviews #24&26)

#### Business Activity Suspension

Business operation was impacted in several ways. For example, limited interactions with employees and facilities closure changed the nature of the workplace. With social-distancing and non-contact regulations and requirements, customer experience was often compromised. Contactless services cannot build close relationships with customers as mentioned by participants. Also, due to government restrictions on mass gatherings, businesses remained vacant or closed. All these factors contributed to revenue loss and with rising costs, many businesses had to resort to operational costs reduction for survival.

#### Employee Layoff and Labor Leakages to Other Industries

Having to reduce operational costs, many tourism businesses resorted to employee layoffs, which contributed to labor leakages to other industries, highlighting a loss of skilled workers and their customer connections. As one respondent mentioned, “businesses had to cut off 30%−50% of employees for survival.” Policies such as “restructuring and redundancy” imposed on airline companies led to significant job loss. The long-term damage will be the loss of skilled workers to other industries.

### Opportunities of COVID-19 for Tourism Businesses

#### Hygiene and Health Safety Improvement (Crisis-Specific)

The pandemic has generated higher health consciousness and awareness in tourism businesses. Since overall travel confidence of visitors has dropped due to health concerns, improvements on hygiene standards and health/safety requirements have been pushed forward industry wide. These have morphed into a new normal/standard operating procedure, which provides a basis for market competition and as a way for better industry and tourism business preparedness for future health crises, as mentioned by participants from different sectors (see Table 2 for full quotes).

#### Sustainability Thinking and Industry Transformation

The pandemic has provided tourism businesses an opportunity to review, rethink, and reevaluate their future plans as reflected in three sub-themes: environmental sustainability, value and process review, and industry reform. First, tourism businesses seemed more concerned about sustainability and over-tourism. With changing travel patterns and visitor behavior due to COVID-19, businesses were more willing to address environmental sustainability issues in the future. Second, participants mentioned how operational problems in the industry have been magnified due to COVID-19. They mentioned the current unhealthy competition among stakeholders in retaining customers and price competition as problematic and needed to be reviewed and changed. This could possibly foster more positive collaboration among stakeholders in future crises, allowing businesses to focus on joint value creation and share a common vision for the tourism industry (see Table 2 for full quotes). Furthermore, some business operators expected a positive transformation of the industry following the pandemic. As one participant mentioned, the old management system in the aviation sector should be renewed and rescheduled, staff recruitment and retention strategies should be refined, and the monopoly in the sector should be disestablished.

#### Market Diversion and Product Innovation

Due to international travel restrictions, tourism businesses were relying on the domestic market (either utilize existing or developing new resources to tap in this market). This required restructuring their target markets and marketing strategies for business survival and continuity. For example, due to many airlines cancelling flights to and from Bangladesh, there was an increase in domestic tourist traffic in many prominent tourist spots (e.g., Coxs Bazar, Sylhet) which counteracted the government lockdown measures. Hospitality and tourism businesses catered for this small but high value segment of domestic tourists who were ignoring lockdown measures and travelling. Furthermore, restaurants found new opportunities to update their business model, such as cloud kitchen and home delivery food services. This accelerated the adoption of new technology and delivery service partners (e.g., online platform technical companies and rider companies) and the development of new collaboration partners to respond to the pandemic.

### Utilization of Dynamic Capabilities for Organizational Resilience

With the 13 resilience indicators representing three categories *(leadership & culture, networks & relationships, and change ready)* of factors and two resilience dimensions (planned and adaptive), Table 1 illustrates the themes and sub-themes that were derived from the data. The respective action themes and corresponding DCs in different crisis phases to structure various resilient elements are presented in Table 4. Categorizations of DCs for each crisis management phase are drawn from the 12 resource-based typology of DCs (Jiang et al., 2021a).

The results highlight low preparedness among tourism businesses before the crisis with only two types of DCs, replicating and integrating DCs, being employed for business continuity. In comparison, a more active use of DCs for different resilience-building activities was noted in the short-term response and potential future long-term recovery actions/strategies, as discussed below.

#### Crisis Preparedness Phase—Readiness

The crisis preparation phase involves actions undertaken to prevent or mitigate the effects of potential crises/disasters (Faulkner, 2001). In this phase, risk assessment and scenarios development are undertaken to predict the potential impacts and prepare for a contingency plan. However, due to the unique nature of the COVID-19 pandemic, most tourism businesses were ill prepared due to: *(i) low situation awareness, (ii) underestimating* COVID-19 *spread and magnitude, and (iii) low expectations of long-term impacts.*

First, many business operators/managers considered the pandemic as having impacts beyond their control and, thus, engaged in limited or no preparation to mitigate impacts. Some businesses had developed a disaster management policy for standard operating procedures based on previous disruptive events (e.g., terrorism and disasters), but this had little application to COVID-19, indicative of low situation awareness. Furthermore, business operators/managers had underestimated the spread and magnitude of COVID-19. Third, the long duration of COVID-19 was not predictable nor controllable. Most businesses expected a 1 to 2-months crisis and had no plans in place to mitigate long-term impacts.

Despite low preparedness, a few tourism operators had utilized *“replicating DCs”* to leverage critical information from past experiences and *“integrating DCs”* to develop contingency plans and reorganized products for crisis readiness. However, these DCs only relied on internal resources, with limited access and posture to acquire external resources in the preparation phase.

#### Replicating DCs (Leveraging Knowledge)

While preparedness for this global pandemic was challenging, some businesses had utilized previous experiences with crisis and disaster management to design response and recovery plans, which is a type of *replicating DCs* to duplicate resources and processes for knowledge transfer management:

Nobody was prepared. But US-Bangla airlines had gathered experiences to face any disaster or crisis management… our previous experiences had helped us to move forward into the future. (Interview #21)

#### Integrating DCs (Proactive Posture and Planning Strategies)

For other businesses that relied significantly on the Chinese market, precautionary actions and contingency plans were developed to maintain business continuity. Constructive decision-making is a key capability to be change ready, which required *integrating DCs* that actively recognize available internal resources and integrate them with existing operating procedures to buffer negative impacts and maintain business continuity.

We took precautions to maintain operations as people were coming from China… when COVID was not even found in Bangladesh. We used to measure the temperature of our guests, kept a log, and monitored them with our house doctor if they had a temperature. We had hand sanitizer at every entry point of our hotel. So, we had our preparation in. (Interview #02)

Furthermore, with *integrating DCs*, some business operators/managers undertook a more comprehensive pre-crisis planning by reorganizing their products and business models before COVID-19 hit the country:

We started making some initial plans about how we will manage our operations and cost during the bad time that was about to come. We have also secured aspects of our business according to the plans by offering our rooms [as home office] for senior employees of banks and factories that were still running. So, we planned something like this for a minimum profit. (Interview #04)

#### Crisis Short-Term Response Phase—Mitigating Impacts

The crisis response phase is usually short-term with a focus on restoring services and moving the tourism industry back to normal operations (Faulkner, 2001). In this phase, businesses usually start to rebuild customer relationships, reconnect with the workforce, and coordinate active communication with partners and other stakeholders. Based on Jiang et al. (2021a), all four types of DCs in the response phase were found among tourism businesses during COVID-19 to maintain business continuity and improve adaptive capacity.

***Reconfiguring DCs (change operational procedures by redeploying existing resources)*** were the most used DCs. They were widely applied to boost adaptive resilience, specifically through leadership, staff engagement, effective partnership, internal resources, unity of purpose, and planning strategy. For example, staff wellbeing and health management were key in the response phase to maintain business continuity and customer relationships. Due to social distancing requirements, more online channels were created to support staff (either from colleagues or employers). These included not only financial support but also reconnecting the workforce to the company:

Our employees are trying to raise a fund for those who are seriously affected. There is a WhatsApp group through which everyone can know each other which is a positive thing to me. (Interview #04)

In addition, business operators/managers had the challenging task of maintaining operations with low revenue. Many chose to reduce operational costs by rearranging their existing resources, for example minimizing food costs, renegotiating vendor contracts, closing venues, and shifting employee working hours.

#### Creating DCs (Exploration for New Business Values by Recombining Existing Resources)

In contrast to disasters (Jiang et al., 2021a), creating DCs were more widely utilized as a response to the pandemic for resilience-building. For example, new emergency team development and health monitoring systems were implemented by tourism businesses to respond to the unique context of COVID-19. In some hotels, an emergency team was established to look after guests and communicate with authorities until the situation went back to normal. Furthermore, a health monitoring system was created for staff members to ensure workers’ health and safety and as a health record for changing work arrangements:

We already placed a health monitoring system for our colleagues. People are required to fill the questionnaire every week that is set in our google doc. (Interview #05)

***Assimilating DCs (absorb external resources for effective response)*** were utilized to find external resources to support employment, acquire learning experience from other businesses, and absorb new technologies to adapt to the “new normal.” For example, some businesses were actively seeking funding from government and NGOs to support employees. This awareness and engagement with external resources were important for short-term response when internal resources were either unavailable or insufficient. Furthermore, external collaboration and coordination were important for clear and consistent planning strategies within the sector or the wider industry:

Bangladesh hotel and Guesthouse Owners Association, Bangladesh International Hotel Association, Tour operators Owners Association, these associations are planning and working together and making a guideline to adapt to the situation. We are trying to communicate with all our business clients and investors to run our business as much as possible. Interview #20)

#### Developing DCs (Development of New Resources)

Developing DCs were utilized to support staff and new partnership development during the crisis. First, online training was developed by some tourism businesses to foster staff self-development and included COVID-19 related health training as well as free certification courses to upskill employees during lockdowns:

For some senior employees, I have recommended some online free certification courses. Everyone in the management team is doing something to build other skills like presentation skills. I have 14 people in my management team. I had given the courses to everyone and almost 70% of them have already completed them. (Interview #17)

Moreover, smart businesses can develop new operation opportunities by utilizing external networks and partnerships. Despite the widespread suspension of civil airlines, some businesses shifted their focus to cargo flights to maintain regular business operation. To achieve this, exploration of available opportunities from the wider industry was important to create a new business model. External networking, positive business experiences based on past interactions, and trust were critical to secure collaboration with public sector organizations:

We got permission from the government to operate cargo flights for this emergency time. The cargo compound of these aircrafts can carry up to 15 ton without passengers. We took this initiative in the first place and applied to the government for this. They observed our business practices, past records and gave us the chance to operate. (Interview #21)

#### Crisis Long-Term Future Recovery Phase—Adapting for the Future

The crisis recovery phase is long-term when the focus shifts to activities that involve seeking new opportunities and directions for businesses and possible industry transformation (Ritchie & Jiang, 2019). In this phase, a reflection on business practices and active learning can take place after crisis reassessment to restore or reconsider new routines for an improved state establishment (Faulkner, 2001). At the time of data collection, Bangladesh was still in the early phase of the pandemic and, thus, the recovery activities described below are both immediate and long-term. Three types of DCs were found in this recovery phase. Innovation DCs were less adopted due to limited availability of new external resources that could be acquired and exploited by businesses for sustainable development.

***Adaptive DCs (strategically link existing resources within business)*** were widely utilized in achieving immediate adaptive resilience, which included staff engagement, effective partnership, unity of purpose, and planning strategies. Yet, some businesses were acutely aware of having to develop more effective partnerships, new business-customer relationships, and improve communication strategies to better serve customers post-crisis but also as a way to manage changing health risk perceptions in society at large. These were more long-term adaptive practices intentions. Apart from developing active virtual communication with customers and one-to-one communication, trust-building and financial support were also deployed immediately by businesses to transform existing internal resources into sound customer relationships.

Furthermore, participants recognized that to rebuild customer confidence and trust, new hygiene and sanitation measures (e.g., enhanced cleanliness) had to become the norm for service standards in the “new normal” post-COVID-19. This was not only critical for the accommodation sector (e.g., hotels) but also for the general food and beverage sector (e.g., restaurant) and the aviation sector (e.g., airlines). This implied a routine and service pattern change as a key priority for the tourism/hospitality industry in both the immediate and pending long-term recovery.

***Renewing DCs (internal exploration to refresh and augment resources)*** were adopted more in staff engagement, breaking silos, and planning strategies (new products). First, human resource development was one of the key priorities in the immediate recovery phase. Many businesses in the tourism sector were focusing on staff professional development which will eventually benefit business operations. More online trainings were provided during the pandemic to upskill workforce and upgrade training processes:

We have tried to have online sessions with the rest of the staff as well as to give them a feel of collaboration. Giving more deep ideas about their work, given them work related training as much as possible. (Interview #22)

Second, to deal with financial and business continuity issues, some operators had been trying to negotiate with government about tax and other vendors about costs, aiming to achieve a better structured financial solution for business stability in the future. This is part of the learning process of crisis management when new solutions are required to augment opportunities within the scope of existing resources.

***Regenerative DCs (exploitation of external resources to benefit internal operation)*** were utilized in improving situation awareness and developing effective partnerships. First, new information on health and safety (guidelines and compliance requirements) was needed for business stability. Businesses needed to follow external information and government instructions to restructure operating activities but also to enable accurate communication with customers. Specifically, learning from countries that had already experienced the COVID-19 outbreak was utilized as a way for businesses to plan forward and develop actions:

We are following different predictions by countries like Singapore, given that we are observing a different situation in Bangladesh. (Interview #25)

Second, new or redefined partner/network relationships were needed to capitalize on new business opportunities. Some risky sectors (e.g., aviation) needed to develop better relationships with banks who could then assist with financial support. However, uncertainty related to the future of this sector was also compromising relationship building activities. Businesses needed to rethink or reconstruct personal relationships beyond organizational relationships as part of their recovery:

The bank would have given us [aviation sector] some financial support out of personal relationships, and the amount of financial support would have increased once the business relationship was established. But now even if you are the closest relatives of a bank official, you will not be given any financial assistance.

***Innovative DCs (exploration of external resources to achieve sustainable development)*** were utilized to develop creative solutions and improve sustainable development. For example, a travel agency organization generated a unique new service for customers during the pandemic to improve their safety and offer a better experience. This new service was enabled by building new external connections with embassies/airlines, which could then also contribute to strengthening customer relationships for the future:

Some of the services that we are rendering now which we didn’t do in the lifetime of our company is offering\arranging transporatation\logistic services to foreign nationals who are stuck in Bangladesh. We are connecting them with different embassies and connecting with different chartered airlines so that they can go to their own country. (Interview #12)

### Differences in Resilience at Sector Level

We also ran a matrix coding in NVivo to investigate potential differences between planned and adaptive resilience at sector level. We found a similar pattern of responses across tourism, hotel, restaurant, and aviation sectors. Overall, planned resilience *(change ready)* was lacking but more dominant in the responses than adaptive resilience *(leadership & culture, networks & relationships)* at all stages of the crisis. As noted previously, in the crisis preparation stage, “no preparation” was common for all sectors. In the same stage, the hotel and tourism sectors had implemented more *proactive posture strategies* than *planning strategies*, while little was achieved on other resilience aspects. In the short-term response stage, the hotel sector was more focused on *recovery priorities* and *staff engagement*, while both the restaurant and aviation sectors highlighted *planning strategies* more often than *recovery priorities*. In the future long-term recovery stage, all sectors highly valued *planning strategies* aiming to build a more resilient business, such as routine changes, service pattern updates, and product development. Specifically, *recovery priorities, planning strategies* and *external resources* were more important to the hotel sector, while tourism and aviation sectors focused more on *external resources* and *planning strategies*. Restaurants paid little attention to external resources and *proactive posture*.

## Discussion and Implications

Using the DMC, this study identified pandemic threats and opportunities that activated different types of DCs and how they subsequently activated planned and adaptive resilience in tourism-related businesses. The findings, summarized in Table 5, have both theoretical and managerial implications.

**Table 5.** Summary of Findings: Resilience Indicators and Dynamic Capabilities Typology in each stage.

Dynamic Capabilities in Each Crisis Stage Resilience Indicators	Pre-crisis: actions of crisis preparedness	During crisis (short-term response): strategies to mitigate impacts	Post-crisis (future long-term recovery): adaptive practices intentions to recovery
Leadership & Culture (*Adaptive resilience*)			
Leadership		Reconfiguring DC Creating DC	
Staff Engagement and Involvement		Reconfiguring DC Developing DC	Adaptive DC Renewing DC
Situation Monitoring and Reporting			Regenerative DC
Decision Making		Creating DC	
Innovation Creativity			Innovative DC
Networks & Relationships (*Adaptive Resilience*)			
Information and Knowledge	Replicating DC		
Minimization of Silos			Renewing DC
Internal Resources		Reconfiguring DC	
Change Ready *(Planned Resilience)*			
External Resources		Reconfiguring DC Assimilating DC	Regenerative DC Adaptive DC
Recovery Priorities		Reconfiguring DC Creating DC	Adaptive DC
Proactive Posture	Integrating DC		
Planning Strategies	Integrating DC	Reconfiguring DC Assimilating DC Creating DC Developing DC	Adaptive DC Renewing DC
Participate in Exercise			
Others	No preparation		No planning

### Theoretical Implications

Drawing on MRT (Merton, 1968), which considers how context shapes mechanism and outcomes, we identify the importance of different types of DCs in building organizational resilience. DCs are capable of “changing status quo” because their main actions are in relation to “integrate, build, reconfigure, create, extend, and modify” current resources of firms (Teece et al., 1997), which include both assets and capabilities (Jiang et al., 2019). DCs can help organizations to cope with a dynamic and disruptive environment, modifying operating routines and the resource base, while sustaining competitive advantage (e.g., Teece, 2007; Zollo & Winter, 2002), as suggested by the findings of this study. DCs can be distinguished from “ad hoc problem solving,” which is non-repetitive and patterned, given that they facilitate organizations to recover from disruptions (Winter, 2003). However, to build resilience in disruptive environments, ordinary capabilities and reactive problem-solving strategies need to be replaced with higher-order capabilities that support businesses to integrate, reconfigure, review, and recreate their resources and more importantly reconstruct their core capabilities (Jiang et al., 2019; C. L. Wang & Ahmed, 2007). In this way, DCs theory can be linked to organizational resilience in the pandemic context.

Resilience is context based and non-enduring (Jiang et al., 2021b), requiring the activation of different types of DCs in the early stages of the pandemic, as demonstrated in the findings of this study. This departs from existing studies (Alonso-Almeida et al., 2015; Jiang et al., 2021a, 2021c; Shrestha & L’Espoir Decosta, 2023) that do not examine planned and adaptive resilience implications emanating from the utilization of DCs in tourism firms either as part of the response in business-as-usual or disruptive environments. In terms of *context* and aligning with previous studies (Arbulú et al., 2021; Gössling et al., 2021), pandemic threats confirm the financial burden on tourism firms from the first wave of COVID-19, but also the ensuing employment crisis through, for example, employee lay off. The human resource challenges facing the industry at the beginning of COVID-19 identified in this study align with those noted in previous studies (Baum et al., 2020; Gössling et al., 2021; Kaushal & Srivastava, 2021; Sigala, 2020). Operational issues such as business closure and supply chain issues identified in this study conform to impacts noted in other studies (Gössling et al., 2021; Sigala, 2020). Yet, the pandemic also provided opportunities for boosting health and safety protocols in the workplace as suggested by others (Baum et al., 2020) and market transformation that might not endure. However, as highlighted by Price et al. (2022), transformation seems to be driven more by necessity than a strategic shift, although evidence exists on the rising importance of sustainability for these firms in the immediate and long-term future recovery. Thus, transformation is still a future possibility as these businesses ponder on integrating sustainability and resilience practices in their strategic capabilities development.

The *context* shapes the response as tourism firms utilize DCs to improve resilience in the immediate response and recovery but also as part of forward planning. Similar to Jiang et al. (2019), we provide a framework (Figure 1) that highlights how existing operational routines can transform into new DCs that support resilience to disruptive events. From a process view of resilience, our results demonstrate low crisis preparedness of tourism firms, a theme that permeates the organizational resilience literature (e.g., Orchiston et al., 2016; Prayag, 2020; Prayag et al., 2020). More importantly, the use of DCs as a *mechanism* to activate resilience is limited in the pre-crisis stage, which can be partly explained by the development stage of the tourism industry in Bangladesh, with a focus on the domestic market. Thus, resilience is adaptive rather than planned. Evidence of rapid decision-making during the pandemic exists for some of the businesses in this study but several indicators of adaptive resilience are largely absent such as breaking silos and situation awareness (Lee et al., 2013). Their use of leadership and culture is also not apparent, and firms relied primarily on existing networks and relationships to leverage knowledge. Thus, they engaged in limited forward planning.

Similar to Jiang et al. (2021b) we found that in the short-term response, tourism firms engaged in continuous dynamic monitoring of the external environment to shape their response and recovery. They draw on resources, knowledge and capabilities embedded internally and externally to drive mitigation and response. Table 5 shows several types of DCs (reconfiguring, creating, developing, and assimilating) were utilized to extract resilience benefits related to leadership, staff engagement, rapid decision-making, and building effective partnerships. Thus, in relation to RQ1 and RQ2, Table 5 provides evidence of different types of DCs employed at different stages of the DMC. Accordingly, our findings suggest that resilience develops incrementally, creating a continuous feedback loop for future preparedness to crises and disasters (McManus et al., 2008) rather than an initial preparedness to COVID-19. In this way, we extend Jiang et al. (2021b) by linking DCs to planned and adaptive resilience indicators in a staged DMC approach.

DCs seem to facilitate organizations to exploit resources and capabilities to bounce back and move to business-as-usual, while also providing competencies for future growth. As evidenced in the recovery stage (immediate and future), DCs (adaptive, renewing, regenerative, and innovative) allowed tourism firms to create unity of purpose, breaking silos, engage in forward planning and adopt a proactive posture. In this way, tourism businesses through their COVID-19 experience become more change ready for future disruptions. Also, uncertainty drives organizations to use their internal capacities for understanding, evaluating and managing rapidly changing external environments to maintain business continuity (Jiang et al., 2021a) and DCs become a mechanism to achieve this. We found that leveraging knowledge internally allowed organizations to quickly modify markets and products as suggested in other studies (Denford, 2013; Jiang et al., 2021a), while leveraging external networks facilitated the acquisition and transformation of resources, reflecting the essence of DCs (Blyler & Coff, 2003). We also note the lack of innovative DCs driving recovery, suggesting that firms were adopting a “wait and see” approach post-pandemic, possibly due to uncertainties around government policies and their industry support for the longer-term.

Overall, we found support for 10 of the 12 types of DCs mentioned by Jiang et al. (2021a). Two DCs in the crisis preparedness phase were lacking: imitating DCs and synthesizing DCs. This finding illustrates a strong reliance of local tourism and hospitality businesses on “internal resources,” which are either focused on replicating existing knowledge or integrating existing resources into new business processes. The lack of recognition and utilization of external resources (either exploitation or exploration) can be caused by several factors. First, the tourism industry in Bangladesh is not networked tightly and most businesses are working independently with little disaster management experience being shared between businesses, despite the country facing frequent disasters. This highlights low collaboration within the industry for building its resilience. Second, a mind-set of “powerless and unable” to prepare for a pandemic was dominant in the data. Thus, business owners/managers were less motivated to search for external resources in preparing and responding to disruptive events.

From a DMC perspective, a pandemic is very different from other crises and disasters in both its impacts and response from tourism businesses. Disasters have a relatively shorter emergency and response phase (e.g., earthquake and floods) but the recovery and reconstruction phases can take years (Ritchie, 2004). In contrast, the COVID-19 pandemic seems to have a prolonged response phase with many countries maintaining strict control policies for 2.5 years now and recovery is protracted in many instances. Many tourism businesses in our study, however, started to integrate recovery strategies in the early stages of the pandemic and planning future recovery actions and strategies. Thus, they adopted adaptive practices in an early stage compared to other disasters and thought of maintaining and improving these over time. For example, at the time of data collection, staff professional development, tax negotiation with government, and learning from the response of other countries were already in place for some of the businesses. While all of the sub-sectors were weak on planned resilience before the pandemic, they had different priorities and pathways in relation to resilience in the short-term response phase. In particular, adaptive resilience strategies and priorities for future recovery were different, highlighting that sectors within the same industry can approach resilience building differently.

From a methodological perspective, using a staged DMC approach for in-depth interviews provides structure to how participants reflect on organizational practices that are embedded in DCs and resilience thinking. Combining this interviewing approach within an MRT framework, provides the missing link in the organizational resilience literature on how impacts of disruptive events affect the utilization of DCs, which in turn, activate planned and/or adaptive resilience.

### Managerial Implications

First, without DCs tourism organizations struggle to extract resilience benefits from operational activities and networks (internal and external) as a response to the COVID-19 pandemic. Coping with different threats and responding to different stages of the pandemic require more than just sensing, seizing and reconfiguring DCs. Understanding critical levers in the tourism supply chain, for example, which often involves multiple stakeholders (such as government, suppliers, customers, competitors, etc.) who are actively engaged in minimizing negative impacts of the pandemic can facilitate the identification of DCs (reconfiguring, assimilating and creating) that would support resilience building activities. These DCs can identify knowledge and capabilities that can underpin mitigation and response strategies.

Second, tourism managers should build replicating and integrating DCs in the crisis preparedness phase that would allow them to quickly ascertain which existing resources or capabilities should be deployed in the response phase. For example, managers need to allocate crisis related roles and responsibilities to specific employees based on revised standard operating procedures (SOP) when utilizing these two types of DCs. In the case of Bangladesh, the revised SOP can be adapted for tourism firms based on general guidelines provided by government for COVID-19 (see Pratiwi & Mahmudatussa’adah, 2021). To action these guidelines, tourism managers should adhere to safety protocols in their dealings with employees, customers and other stakeholders, providing the baseline to build strategies that are adaptive and responsive to a fast-changing external environment. DCs would also allow future proofing of tourism businesses and highlight opportunities for long-term transformation that is contingent upon the adoption of a more flexible and open-to-change culture during any disruptive event (Jiang et al., 2021c).

Third, the response phase (short-term) requires managers to deploy reconfiguring, creating and developing DCs. This implies the reconfiguration of existing operational procedures (such as customer contact, delivery process, queuing system, etc.) to align with COVID-19 protocols and the development of internal capacities to continuously monitor market changes. Effective utilization of these DCs rests on how quickly tourism firms can get access to pandemic related information, knowledge, and resources (e.g., financial) residing outside of firms. One potential avenue to gain access to such complementary information and knowledge is through nurturing mutually beneficial relationships with existing business partners (e.g., suppliers, customers, competitors) and political partners (e.g., government). Managers can strengthen these relationships through practicing a number of strategic relationship building activities such as organizing social events for business partners and inviting government officials as partners in key employee and industry development programs.

Fourth, long term recovery requires tourism firms to effectively utilize adaptive, renewing and regenerative DCs. This implies changing the nature of existing customer demand, updating existing policy and practices, and diversifying operations. Also, managers need to develop an ambidextrous strategy that focuses on both exploration and exploitation of new opportunities. These can strengthen planned and adaptive resilience. An exploration strategy would concentrate on identifying new opportunities through investing in new IT capabilities (e.g., big data analytic capability) to better understand competitive intelligence. This capability would identify emerging market trends that can facilitate business expansion and diversification. An exploitation strategy, for example, would focus on improving the efficiency of a firm’s use of internal resources such as the development of multi-tasking abilities in employees through new training programs, enhanced use of IT capabilities to better connect with customers, and updating HR policy to facilitate employee retention and wellbeing long-term. These two strategies can create a virtuous cycle of resilience building in tourism organizations during and post-pandemic.

## Conclusion, Limitations, and Areas for Further Research

In conclusion, through the identification of pandemic impacts in Bangladesh, we have uncovered different types of DCs that were deployed by tourism firms in a staged DMC approach to build organizational resilience. As such, our study contributes to extend DCs theory in showing that DCs other than sensing, seizing and reconfiguring were activated during the pandemic to build planned and adaptive resilience of tourism firms. We also use MRT to isolate pandemic impacts on tourism firms from their subsequent influence on DCs and organizational resilience, an issue which the resilience literature (Hillmann & Guenther, 2021) has pointed as being a weakness in existing studies. In these ways, we extend previous studies on DCs in disruptive environments (Jiang et al., 2021b, 2021c; Shrestha & L’Espoir Decosta, 2023). However, our study has several limitations that can be addressed in future research. First, we provided qualitative evidence embedded in participant and business characteristics, and therefore, findings should be considered only as exploratory. Future research can provide quantitative evidence of the relationships between different types of DCs and organizational resilience. Second, not all types of DCs were useful in building resilience during the pandemic, such as imitating and synthesizing DCs during the crisis preparedness stage, which suggest the possibility of firm size affecting the use of different types of DCs. Smaller firms seem to be employing fewer types of DCs than larger firms. Future research can explore the interface of both context (e.g., crises or disasters) and organizational characteristics in determining types of DCs employed for resilience building but also the influence of national culture on business practices.

Third, our study does not consider that DCs in themselves can have reciprocal effects. Future research can extend this by identifying the synergistic effect of these DCs in different phases of crises/disasters. For example, the combined effects of assimilating and creating DCs during the response phase of a crisis can have a stronger impact on resilience. Fourth, while our study assumes through its design and analysis that DCs directly influence organizational resilience, there are instances where other organizational and institutional capabilities can intervene in strengthening or weakening such influences. For example, Jiang et al. (2019) proposed the role of operational capabilities as an effective mechanism that translates DCs into organizational resilience. At an institutional level, governance structures in the tourism industry and tourism policy changes can have a direct impact on organizational resilience, without needing DCs to extract resilience benefits for organizations. These areas could be explored in future studies. Fifth, the male only sample could potentially have an impact on strategic decision making and the types of DCs and resilient practices that are prioritized within an organization and, thus, integrating female and other genders in future studies might highlight different results. Yet, Figure 1 is derived from the literature and would have applicability in other contexts in researching disruptive environments, irrespective of sample composition, albeit differences in prioritizing and implementing practices that can enhance DCs and organizational resilience might emerge. Sixth, data were collected during the early wave of the pandemic and since then response and recovery strategies might have been altered to cope with subsequent waves of the pandemic. As such, DCs and resilience strategies deployed in the early phase might not correspond to those utilized in subsequent waves of the pandemic. Longitudinal studies can ascertain more accurately the types of DCs and resilience strategies that have been effective for tourism firms to navigate the COVID-19 pandemic.

## Appendix A: Interview protocol

1. Please tell us about the broad impacts (positive and negative) of COVID-19 on your business sector? [probe: employment, sector performance, network and relationships, supply chain]
2. What are the impacts of COVID-19 (positive and negative) on your business? [probe: business performance, employment/staff engagement, operations, finance/revenue, market change]
3. Were you prepared at all to face this unexpected disruption? [probe: If yes, how… if no, why not?] How does this compare to planning for other disasters that you might have faced as a business? [probe: any prior disaster plans/preparation activities]
4. What strategies have you put in place since then to limit the impacts of COVID-19 on your business? [probe: operations, staff engagement, networks and relationships, leadership, organizational culture, other proactive strategies taken]
5. Can you give us examples of how your business has adapted to changing circumstances related to COVID-19? [probe: working pattern, finance, marketing, human resources/employment, business processes/procedures, strategic priorities]
6. What have you put in place to bring the business back to some form of stability? [probe: processes, procedures, new initiatives, activities/strategies, new business relationships]
